# The Relationship between Comprehension of Figurative Language by Japanese Children with High Functioning Autism Spectrum Disorders and College Freshmen's Assessment of Its Conventionality of Usage

**DOI:** 10.1155/2013/480635

**Published:** 2013-10-28

**Authors:** Manabu Oi, Sanae Tanaka, Harue Ohoka

**Affiliations:** ^1^Research Center for Child Mental Development, United Graduate School of Child Development, Kanazawa University, B-b43, 13-1 Takaramachi, Kanazawa 920-8640, Japan; ^2^Nihon Fukushi University Chuo College of Social Services, 3-27-11 Chiyoda, Naka-ku, Nagoya 460-0012, Japan

## Abstract

Unlike their English-speaking counterparts, Japanese children with high-functioning autism spectrum disorders (HFASDs) perform as well as typically developing (TD) children in comprehending metaphor, despite lacking 1st order theory of mind (ToM) reasoning. Additionally, although Japanese sarcasm and “indirect reproach” appear theoretically to need 2nd order ToM reasoning, HFASD children without this comprehended these forms of language as well as TD children. To attempt to explain this contradiction, we asked college freshmen to evaluate the strangeness (unconventionality) of these types of figurative language. We aimed to test the hypothesis that metaphor, sarcasm, and “indirect reproach” might be evaluated as more conventional than irony, which children with HFASDs do not comprehend as well as those with attention deficit hyperactivity disorder. The results for irony, metaphor, and “indirect reproach” supported the hypothesis, while those for sarcasm did not. Sarcasm is comprehended by HFASDs children as well as by TD children despite being evaluated as highly unconventional. This contradiction is discussed from a self-in-relation-to-other perspective. We postulate that a new explanation of disabilities of figurative language comprehension in children with HFASDs is needed instead of relying on a single cognitive process.

## 1. Introduction

As Norbury and Sparks [1] have suggested, autism spectrum disorders (ASDs) might be better understood when examined from a cultural point of view. Cross-cultural studies might also help to refine cognitive theories of disorder that have been derived exclusively from North American and European investigations. This is the case for the comprehension of figurative language in autism [2–9], which seems to vary greatly between cultures in terms of ways of using metaphorical expression and saying something disagreeable. For example, in Japanese, metaphors are more fluid than in English [10]. Moreover, while irony is regarded as conveying not just a negative meaning but also humor in English, few studies have attempted to investigate any positive role irony has to play in Japanese conversation [11]. Moreover, Japanese has many varieties of sarcasm [12]. The average Japanese person would find it hard to distinguish between the English terms “irony” and “sarcasm.” Even among professionals, the Japanese *hiniku* is translated not only as “irony” but occasionally as “sarcasm,” as by Adachi et al. [13]. In the present study, irony was defined as “the expression of one's meaning by using words of the opposite meaning in order to make one's remarks forceful.” Sarcasm was defined as “the expression of one's meaning by using words of the opposite meaning in order to taunt the hearer.”

Additionally, in addition to irony and sarcasm, Japanese researchers have coined the phrase “indirect reproach” [14], an expression intended to mitigate a face-threatening act toward the hearer by avoiding direct expression of anger or irritation. “Indirect reproach” has been defined as “criticizing the hearer by referring to any contextual information that relates to the speaker's intention.” English does not contain a counterpart for this type of phrase. Investigating how these Japanese figurative language styles are comprehended in children with high-functioning autism spectrum disorders (HFASDs) seems beneficial for understanding autism from a cross-cultural point of view.

 English-speaking children with HFASDs find irony more difficult to comprehend than metaphor [2, 3]. The same appears true for Japanese speakers. Adachi et al. [13] showed that Japanese children with Asperger syndrome (AS), ranging in age from 7 to 14 years, comprehended irony less well than children with attention deficit hyperactivity disorder (ADHD) matched for IQ and age, while there was no intergroup difference in metaphor comprehension. However, these studies differ in terms of theory of mind (ToM) development in the participants. Unlike the studies of Happé [2, 3], Adachi et al.'s study [13] did not test 2nd order ToM reasoning. Among the latter participants, those who passed 1st order ToM tests performed better in comprehending irony than those who failed such tests [13]. It is probable that the participants of Adachi et al.'s [13] study did not achieve 2nd order ToM reasoning. If this is the case, Adachi et al.'s results might differ from those of Happé [3] with respect to the relationship between ToM and irony comprehension. As for metaphor, Japanese AS children who failed 1st order ToM tests comprehended metaphor well [13], unlike their counterparts in Happé's study [3], which reported that adolescents who did not pass 1st order ToM tests also failed to comprehend metaphor. These differences suggest that, at least in Japanese children with HFASDs, 2nd order ToM reasoning is not necessary for irony comprehension, and that 1st order reasoning is not necessary for metaphor comprehension. This suggests that a factor other than ToM reasoning might influence figurative language comprehension, accounting for the differences between Japanese and English in this respect.

What about figurative language other than metaphor and irony in Japanese? In terms of understanding sarcasm and “indirect reproach,” Yata and Oi [14] investigated children with HFASDs, ranging in age from 8 to 15 years, and typically developing (TD) children matched for age and receptive vocabulary. When these children were presented with five sentences for each type of language (i.e., 10 sentences in total) in the written form, no differences in comprehension were evident between the two groups. In addition to this, Taguchi et al. [15] investigated children with HFASDs, ranging in age from 8 to 16 years, and age-matched TD children. That study had similar findings regarding sarcasm and “indirect reproach” comprehension. 

Oi and Tanaka [16] compared children with HFASDs (ranging in grade from 2nd to 6th) and grade-matched TD children with regard to comprehending ambiguous sentences including metaphor and sarcasm but not irony. They found intergroup differences for only 10 of 50 sentences. These 10 sentences included one metaphor (Appendix) and nine grammatically, lexically, or contextually ambiguous sentences. Of the 10 metaphors, no group difference in understanding was found for nine, and the two examples of sarcasm were understood equally well by the TD and HFASD children. Children with HFASDs showed literalness in only four of the 50 sentences. The most interesting finding was that HFASD children showed overnonliteral comprehension compared with TD children for six contextually or grammatically ambiguous sentences.

In terms of the reason why children with HFASDs fail to comprehend figurative language, Happé [3] postulated that without 1st order ToM reasoning they could not understand metaphor, and without 2nd order reasoning they would fail to comprehend irony. So how do Japanese forms of sarcasm and “indirect reproach” compare in this regard? These two forms of language appear similar to irony in terms of requiring some metacognitive ability for comprehension. Awareness of thought might be required on the part of children, meaning that they would need 2nd order ToM reasoning to comprehend these two types of language. An example of a sarcasm task given by Yata and Oi [14] was to choose a response describing the mother's opinion of the child for the following scenario: a mother said, “You're a genius, aren't you?” to a child who got a very bad mark in an exam (see Appendix for detail). An example of an “indirect reproach” task given by Yata and Oi [14] was to choose a response describing the boy's opinion of his friend in the following scenario: a boy said, “Are you leaving without tidying up?” to his friend who was getting ready to go, leaving a mess behind (see Appendix for detail). In addition, Japanese metaphors were again comprehended by children who failed to demonstrate 1st order ToM reasoning in this study [14] while English ones were not understood by their English-speaking counterparts in Happé's study [3].

Why were English ironic phrases not understood by children with ASD while Japanese sarcasm and “indirect reproaches” were, despite the fact that these three forms of figurative phrase appear to equally need 2nd order ToM reasoning? Of 20 children with HFASDs investigated by Yata and Oi [14], nine failed the 2nd order ToM task and three failed even the 1st order ToM task. Why were these children without 2nd order ToM reasoning able to comprehend sarcasm and “indirect reproach”? Additionally, why could Japanese metaphors be comprehended by those showing no 1st order TOM reasoning, while English ones were not understood by their English-speaking counterparts? Japanese and English seem to differ greatly from each other in terms of figurative language comprehension from the viewpoint of ToM. This reminds us of the theory by Hinds [17] that Japanese is a listener-responsible language while English is a speaker-responsible language. With less responsibility to make the message as clear as possible for the hearer, speakers of Japanese could rely more on figurative language than those of English. This might uniquely influence the development of figurative language comprehension in Japanese children with and without HFASDs when compared with their English-speaking counterparts.

Thus, another explanation for these differences should be sought other than the development of ToM reasoning. One possible factor is the role of conventionality or salience [8] in comprehending figurative language. The most suitable example of this in English comes from Ozonoff and Miller [4]. In investigating humor, inference, and indirect request comprehension in adults with autism and preserved intelligence quotient, they showed that “Can you …?” type questions were comprehended more nonliterally by adults with autism than by matched controls. They argued that individuals with autism have overlearned the rule that questions beginning with “Can you …” should be interpreted in a nonliteral way. In everyday situations, such syntactic forms are more likely to be polite requests for action than inquiries about ability. Ozonoff and Miller [4] assumed that individuals with autism may be less able to use context to determine when this rule should not be applied, due to difficulty inhibiting familiar or overlearned responses. This might relate to a deficit in executive function. 

In any case, it is worth evaluating figurative language tasks in terms of their conventionality in order to better explain why Japanese sarcasm, “indirect reproach,” and metaphor were successfully comprehended by children with HFASDs, irrespective of the level of ToM achieved. Statements in these three categories might be evaluated as more conventional when compared with irony. This hypothesis is plausible because Giora et al. [8] showed that “the graded salience hypothesis” could explain why their participants with AS responded to metaphorical and literal language in a similar way to that of controls. According to their study, both the AS group and controls performed worse on novel expressions than on familiar ones, whether literal or metaphorical. The novelty and familiarity of expressions were defined operationally by the authors. In the present study, we instead determined the degree of novelty of figurative language by having it evaluated by college freshmen, like Oi and Tanaka [18] did in showing that the comprehension of ambiguous language tasks in children with HFASDs was highly correlated with the evaluation of the sentences in terms of conventionality by college freshmen. The correlation (*r*) between children's mean literal-nonliteral preference magnitude (comprehension) and freshmen's mean strangeness rating (unconventionality) was −0.65 (*P* < 0.001) for children with HFASDs and −0.67 (*P* < 0.001) for TD children. The two *r* values did not differ significantly from each other.

 The present study tested the abovementioned hypothesis that metaphor, sarcasm, and “indirect reproach” would be evaluated as more conventional than “irony” by asking college students to evaluate from the viewpoint of conventionality examples of these forms of language previously given to children with and without HFASDs. We compared the conventionality evaluation among these four categories of figurative language. 

## 2. Materials and Methods

### 2.1. Participants

University freshmen were recruited in random order. Participants were 98 male freshmen (mean age = 19.50 years, SD = 0.74) and 96 female freshmen (mean age = 19.19 years, SD = 0.64). 

None of the freshmen were evaluated for autistic personality traits. They were asked to rate the strangeness (unconventionality) of figurative language assigned to children. The study was conducted in accordance with Declaration of Helsinki (1964).

First, the freshmen were asked to rate the strangeness of 10 combinations of a written sentence and written scenario in which the sentence was embedded; these had previously been used in Adachi et al.'s study [13] (see Appendix). Of the 10 sentences, five were ironic phrases and the remaining five were metaphors, all of which had been presented to 123 children. These children were diagnosed according to the Diagnostic and Statistical Manual of Mental Disorders, Fourth Edition (DSM-IV), as having AS (*n* = 66, 57 boys and nine girls, mean age = 9.8 years, and SD = 2.0), high-functioning autism (HFA) (*n* = 20, 17 boys and three girls, mean age = 9.4 years, and SD = 2.0), or ADHD (*n* = 37, 33 boys and four girls, mean age = 9.4 years, and SD = 1.8). Mean full-scale IQ as measured by the third edition of the Wechsler Intelligence Scale was 98.6 (SD = 14.0), 93.4 (SD = 12.8), and 98.2 (SD = 14.7), respectively, for each group. The written sentences were presented to the children who read them and were then asked to choose one of five answers to a question based on the sentences. The answers consisted of literal, nonliteral, irrelevant, and situational responses as well as a response indicating that the child had not understood the question.

Second, the freshmen were asked to rate the strangeness of another 10 combinations of the written sentences and scenarios given by Yata and Oi [14] (see Appendix) in which the sentence was embedded. Of these 10 sentences, five were sarcastic phrases and the remaining five were “indirect reproaches.” They had been presented to 20 children with HFASDs diagnosed according to DSM-VI criteria (17 boys and three girls, mean age = 11.99 years, and SD = 2.08) and 20 TD children (13 boys and seven girls, mean age = 11.35 years, and SD = 2.71). These were matched for raw scores in the Picture Vocabulary Test, for which the mean score for HFASDs children was 61.7 (SD = 6.0) and that for TD children was 59.0 (SD = 7.4). There was no intergroup difference in either age or raw score of the test. These HFASD children ranged in full-scale IQ from 71 to 129 (mean = 93.9, SD = 13.4), when assessed using the third edition of the Wechsler Intelligence Scale within a year before the data collection. Children were asked to read the sarcasm and “indirect reproach” scenarios and to answer a question based on these scenarios by choosing one of three options consisting of literal, nonliteral, and situational answers. In addition to this, 10 more combinations of the written sentences and scenarios given by Taguchi et al. [15] were rated by the freshmen. Similarly, of the 10, five were sarcastic phrases and the rest were “indirect reproaches.” These had been presented to 17 children with HFASDs (14 boys and 3 girls, ranging in age from 8.67 to 16.08 years; the mean and SD were not obtained) and 15 TD children (13 boys and 2 girls, ranging in age from 7.08 to 16.33 years; mean and SD were not obtained). 

Third, the freshmen were asked to rate the degree of strangeness of 20 combinations of a sentence and a cartoon picture representing the nonliteral and literal interpretations of the sentence (see Appendix). These sentences were all metaphors. These had been given by Oi and Tanaka [16] to 2nd to 6th graders with HFASDs (40 boys and five girls, mean grade = 4.29, and SD = 1.27) and 45 TD children matched exactly for grade and gender who were extracted in random order from 666 2nd to 6th graders at an elementary school. The TD children all attended regular classes, received no special educational services, and had no sensory or motor impairments. All the HFASD children were assessed by psychiatrists or pediatricians as fulfilling the criteria for at least one of the pervasive developmental disorders of DSM-IV-text revision (TR). They also all attended regular classes. All HFASDs children were assessed using the third edition of the Wechsler Intelligence Scale within a year before the data were collected: full-scale IQ ranged from 79 to 129 (M = 97.56, SD = 17.54) and verbal-IQ from 80 to 136 (M = 98.87, SD = 17.81). No standardized intelligence scale was administered to TD children, because this is not normal practice among typical council elementary schools in Japan. Children were asked to use a five-point scale to indicate whether they agreed with the literal or nonliteral interpretation of the cartoon. A summary of the abovementioned studies is shown in Table 1.

### 2.2. Procedures

The freshmen rated all the figurative language phrases listed above on a five-point scale. 

The strangest (most unconventional) was assigned a score of 5 and the least strange (most conventional) a score of 1. First, we compared strangeness (unconventionality) values among ironic phrases, metaphors (from Adachi et al. [13]), sarcasm, and “indirect reproach” (from Yata and Oi [14] and Taguchi et al. [15]) using the Wilcoxon Signed Ranks test. For this comparison, and that described below, the test was two-tailed, with *Z* values used to calculate *P* values. Second, for Oi and Tanaka's [16] data, we made a comparison between one metaphor (Appendix), in which children with HFASDs preferred the literal interpretation more frequently than did TD children, and the remaining nine metaphors in which no intergroup difference was found. The freshmen's strangeness evaluation of the former metaphor was compared to that of the latter metaphors using the Wilcoxon Signed Ranks test.

 Direct associations between freshmen's strangeness evaluation and children's interpretation of each figurative phrase could not be assessed as the authors of the present study did not have access to the full data of Adachi et al. [13]. Therefore, in the present study, indirect associations between freshmen's strangeness evaluation and children's ability to interpret figurative phrases were analyzed.

## 3. Results

**Figure 1:**
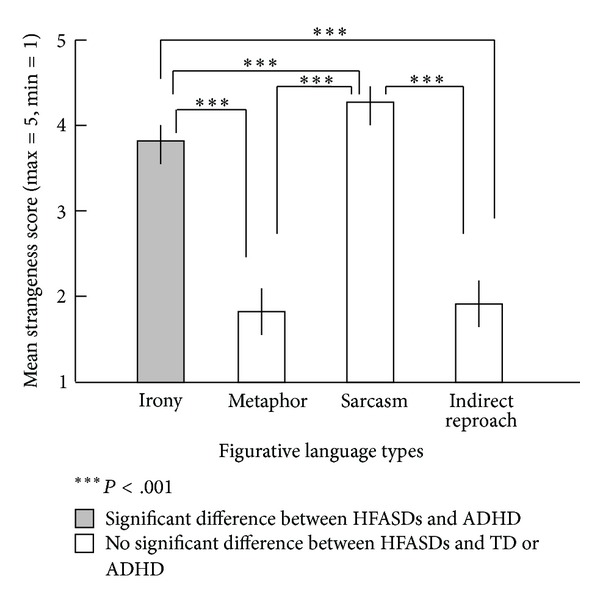
Mean evaluation values of strangeness (unconventionality) by college freshmen for each figurative language type.

As shown in Figure 1, ironic statements were rated as stranger (less conventional) than metaphors (Wilcoxon Signed Ranks test: *Z* scores were applied to calculate *P*; *z* = −11.812, *P* < 0.001) and “indirect reproaches” (*z* = −11.829, *P* < 0.001). Sarcastic phrases were rated as stranger (less conventional) than metaphors (*z* = −12.047, *P* < 0.001), “indirect reproaches” (*z* = −12.048, *P* < 0.001), and ironic statements (*z* = −5.290, *P* < 0.001). No difference was found between metaphors and “indirect reproaches.” For the 10 metaphors from Okamoto [11], the one in which children with HFASD preferred a literal meaning more frequently than did TD children was not rated differently from the remaining nine metaphors.

## 4. Discussion

The results did not fully support the hypothesis that the less strange a figurative language statement was rated, the more easily it would be comprehended by children with HFASD. The results for the ironic statements support the hypothesis as these statements were evaluated as highly strange by college freshmen and were more difficult for children with HFASDs than for those with ADHD to comprehend [13]. The findings for metaphor and “indirect reproach” also supported the hypothesis as both types of statements were evaluated as less strange and were comprehended as well by children with HFASDs as by their TD counterparts [14, 15]. The exception was sarcasm. Although sarcastic phrases were evaluated by the college students as the most strange among the four types of figurative language, these phrases were comprehended as well by children with HFASDs as by TD children, despite the lack of the 2nd order ToM reasoning in nearly half of the children with HFASDs [14]. 

Two questions arise here. The first is why only irony was poorly understood by Japanese children with HFASDs while the other three types of language were comprehended. The ironic statements investigated in the present study were evaluated as highly strange (far less conventional than typical statements). Giora et al. [8] postulate that conventionality can be seen as a major determinant influencing figurative language comprehension both in children with HFASDs and in TD children. They contend that making sense of nonliteral language relies on the salience of that language. According to their graded salience hypothesis, novelty (in other words, unconventionality) matters rather than nonliterality. This appears to hold true regarding the high degree of strangeness of ironic statements in the present study,r which were poorly understood by children with HFASDs, and it is also borne out by the low degree of strangeness of metaphors and “indirect reproaches,” both of which were understood by children with HFASDs.

The present findings regarding sarcasm, however, do not support the graded salience hypothesis at all. We need an explanation that applies to both failure to understand ironic statements and ability to comprehend sarcastic ones. A closer look at the ironic statements used by Adachi et al. [13] might lend some insight into this. Of the five ironic statements investigated, four are not addressed directly from one character to the other in the scenario. Rather these were told in the form of a soliloquy with a humorous feel. In addition to this, in the remaining ironic statement, the child was asked to assume he or she was directly given the statement from another character in the scenario. In contrast, all the sarcastic statements investigated were addressed from one character to the other in the scenario. To succeed in comprehending these five ironic statements, children had to put themselves in the shoes of the character that made the statement or received the statement, while such a need did not exist for the sarcastic statements to be comprehended. In this regard, the metaphors and “indirect reproaches” investigated were similar to the sarcastic statements in that the children did not need to put themselves in the place of the listener or speaker. Hence, in metaphor, sarcasm, and “indirect reproach,” it seems that the child would have understood the statement when he or she could observe correctly what the scenario depicted.

The second question to be addressed is whether or not sarcasm and “indirect reproach” are of sufficient difficulty as to require 2nd order ToM reasoning to be understood. Happé [19] suggested this was the case, as she, based on the findings of Sperber and Wilson [20], made a distinction between an utterance that requires elucidation of “an interpretation of an attributed thought or a desirable thought” and one that requires interpretation of “a description of an actual state of affairs or a desirable state of affairs.” She stated that the former type of utterance includes ironic statements and interrogatives, and the latter includes ordinary assertions and basic imperatives. Happé [19] predicted that the former requires 2nd order metarepresentation while the latter requires only 1st order metarepresentation. The sarcastic statements and “indirect reproaches” investigated here seem to demand the child to interpret the speaker's thoughts regarding the hearer in the scenario. If this is true, how can we explain the finding that nearly half of the participants of the studies of Yata and Oi [14] failed the 2nd order ToM task?

One plausible explanation is again the absence of a need for the child to put her/himself in the shoes of another character to comprehend the statement. Hence, without requiring 2nd order ToM reasoning, children could comprehend sarcasm by logical computations such as those postulated by Oi and Tanaka [18] in learning to recognize situations where people “do not mean what they say.” To do this, children would use simple rules such as literally false or puzzling speech + smile = joke, or literally false or puzzling speech + frown = sarcasm. The sarcastic phrases investigated in the present study were exchanged between third parties for the child. The child could accordingly behave as just an observer of the scenario who computes the meaning of the figurative language. If children had not achieved 2nd order ToM reasoning and sarcasm was not very familiar to them, they could comprehend these scenarios “correctly” by the sort of computation mentioned above, rather than “appropriately” by putting themselves in the place of the character in the scenario. However, children who had achieved 2nd order ToM reasoning could of course use this rather than the more difficult computation. On the other hand, as suggested by the present findings, “indirect reproach” might be more familiar than sarcasm, so that the child has the choice of relying on retrieving the memory of the meaning of “indirect reproach” as well as computation regarding the third parties, instead of using 2nd order ToM reasoning for comprehension.

Taguchi et al. [15] found, although not for the four categories of figurative language investigated here, that children with HFASDs failed to respond to indirect requests appropriately when these were directly addressed to them from adults, yet they succeeded when asked to choose an appropriate response from three types of responses when an indirect request scenario was written or played on a screen. This indicates that the difficulty relates to self-awareness, particularly the need to put oneself in another's place in the scenarios with embedded ironic statements. In responding appropriately to indirect requests such as “Is your mother there?” (meaning “Call your mother”) via telephone, children have to put themselves in the shoes of the speaker. Children with HFASDs, indeed, failed this task [16]. We should therefore take a self-in-relation-to-other perspective [9] when investigating figurative language comprehension in autism, as well as considering ToM, conventionality (salience), weak central coherence, or executive dysfunction (the latter two factors were not discussed in the present study).

Comprehension of figurative language does not consist of a single cognitive process, but should instead be thought of as a product of complex activities of various sociocognitive processes.

## 5. Conclusion

Findings of studies on Japanese children with HFASDs suggest that the development of ToM reasoning is not the sole determinant of figurative language comprehension. The conventionality of figurative language also seems to influence comprehension. The conventionality of figurative language can be measured by having college freshmen evaluate its strangeness (unconventionality). Our findings suggest that Japanese ironic statements were difficult for children with HFASDs to comprehend as these were evaluated as far more strange than metaphors and “indirect reproaches.” In addition to this, Japanese ironic statements seem to require children to put themselves in the position of a character in the scenario. The combination of this with high strangeness may explain why such statements were difficult to comprehend for children with HFASDs. For Japanese sarcastic phrases alone, conventionality did not appear to matter, as these were well comprehended by children with HFASDs despite (like ironic statements) being evaluated as far more strange than metaphors, “indirect reproaches,” or ironic statements. This can be explained by neither the developmental level of ToM reasoning nor the degree of strangeness (unconventionality), but from the fact that the sarcastic phrases were exchanged between third parties. This might help children observe the figurative language and the scenario in which the language is embedded, leading to correct computation of the meaning. When comparing Japanese and English in terms of figurative language comprehension, we require a new way of explaining disabilities in figurative language comprehension as emergent products of complex interactions among sociocognitive activities [21].

 The findings of the present study merit further investigation that directly examines the relationship between children's comprehension and freshmen's evaluation of strangeness of figurative language.

## Figures and Tables

**Figure 2:**
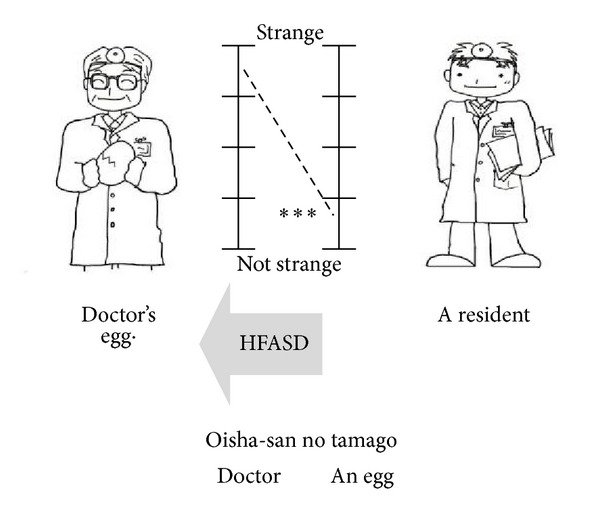


**Table 1:** Summary of the studies by Adachi et al. [13], Yata and Oi [14], Taguchi et al. [15], and Oi and Tanaka [16].

	Number of children	ToM 1st	ToM 2nd	Tasks	Results
Adachi et al. [13]	66 AS children	15 failed	—	5 ironic statements 5 metaphors	Difference between AS and ADHD in comprehension of irony
	20 HFASD children	7 failed	—		
	37 ADHD children	4 failed	—		

Yata and Oi [14]	20 HFA children 20 TD children	3 failed 1 failed	9 failed 8 failed	5 sarcastic statements 5 indirect reproaches 5 indirect requests	No intergroup difference in comprehension of sarcasm, indirect reproaches, or indirect requests

Taguchi et al. [15]	17 HFASD children 15 TD children	— —	— —	5 sarcastic statements 5 indirect reproaches 5 indirect requests	No intergroup difference in comprehension of sarcasm, indirect reproaches, or indirect requests

Oi and Tanaka [16]	53 HFASD children	—	—	10 metaphors	Intergroup difference in comprehension of 1 metaphor
	50 TD children	—	—		

## Appendices

### A. Examples from Adachi et al. [13]

#### A.1. Irony

When Jiro's mother got home, she saw clothes left on the floor of Jiro's room. As she looked at this, she said, “Jiro always leaves his room in a tidy state.” Does the mother think of Jiro as

- a boy who is organized,
- being messy,
- being a boy,
- taking a bath,
- I do not know.

#### A.2. Metaphor

A boy called Goro always wins sprint races. Taro, another boy, while watching Goro leaving the other runners behind, said, “Look, Goro looks like a cheetah!.” Taro thinks of Goro as

- being a cheetah,
- being handsome,
- being a fast runner,
- leaving other runners behind,
- I do not know.

### B. Examples from Yata and Oi [14] and Taguchi et al. [15]

#### B.1. Sarcasm

When a boy got a very bad mark in an exam, the mother told him, “You're a genius, aren't you?”, while looking at the exam paper. What did the mother actually want to communicate?

- Her son is a genius.
- Her son is not a genius at all.
- Her son got a bad mark in an exam.

#### B.2. Indirect Reproach

When a friend of a boy was about to leave a mess behind after having played with many toys, the boy told his friend, “Are you leaving without tidying up?” What did the boy actually want to communicate?

- His friend is leaving now.
- His friend has a very bad attitude.
- They have played with many toys.

### C. An Example from Oi and Tanaka [18]

See Figure 2.
